# The effect of squats on muscle activity in standing, kneeling, and half-kneeling positions: A cross-sectional study

**DOI:** 10.1097/MD.0000000000039902

**Published:** 2024-10-04

**Authors:** Taewoong Jeong, Yijung Chung

**Affiliations:** a Soonchunhyang University Bucheon Hospital, Bucheon, Republic of Korea; b Department of Physical Therapy, College of Health and Welfare, Sahmyook University, Seoul, Republic of Korea.

**Keywords:** electromyography, hip, knee, muscle activity, squat exercise

## Abstract

Kneeling and half-kneeling training are common positions used in physical therapy. however, research on lower extremity muscle activity is lacking compared to the ergonomic aspects and trunk muscle activity. The purpose of this study is to investigate the effects on lower extremity muscle activity during squats in kneeling and half-kneeling positions. The study was designed as a cross-sectional study with a single group of healthy adults. Participants were instructed to perform squats while assuming 3 positions (standing, kneeling, and half-kneeling). Surface electromyography data were recorded 3 times from the rectus femoris (RF), gluteus maximus (GMax), gluteus medius (GMed), and biceps femoris (BF) on the participant’s dominant side, and the mean values were analyzed. The participants performed squats for 9 seconds, with 4 seconds of the descent phase, 1 second of the maintenance phase, and 4 seconds of the ascent phase. A metronome was used to ensure precise timing. The study included 30 participants (19 males and 11 females). The muscle activities of the RF, GMed, and BF showed statistically significant differences among the 3 positions, being highest in the half-kneeling position (HKP), followed by the standing position (SP) and kneeling position (KP). The muscle activity of the GMax was significantly higher in the HKP than in the SP and KP (*P < *.05). The co-contraction ratio was significantly higher with KP than with the SP and HKP (*P* < .05). In the SP and KP, there were statistically significant differences between the ascent and descent phases of the RF, GMax, GMed, and BF (*P* < .05). In the HKP, there were statistically significant differences between the ascent and descent phases of the RF, GMax, and GMed (*P* < .05). The results of this study indicate that squats in the HKP (especially during the ascent phase) require the highest muscle activity, whereas squats in the KP (especially during the descent phase) can be performed with the lowest muscle activity. It can be concluded that these findings could serve as selective indicators for squat exercises and in enhancing postural control, muscle strength, and lower extremity stabilization.

## 1. Introduction

Kneeling is used in various environments and situations.^[1]^ Knee training, including standing in the kneeling position (KP), standing in the half-KP (HKP), stepping from the KP, and kneeling gait, is commonly used in physical therapy.^[2]^ This training is known to establish and reinforce appropriate synergy effects for standing up^[3]^ and is introduced as an intervention to prepare for walking^[4]^ and as an adjunct treatment for transitional patterns^[5]^ in many textbooks.

To maintain standing balance in healthy adults, 2 known selective approaches are the ankle and hip strategies.^[6,7]^ However, in the KP and HKP, the ankle strategy cannot be used, and 1 must rely on the hip strategy to maintain an upright posture. As a result, KP and HKP require trunk and hip control.^[2]^ Furthermore, the knee flexors and extensors require different environments and conditions in the KP and HKP compared to the standing position (SP) or sitting position, potentially requiring stabilization of the thighs and pelvis in relatively unstable positions.^[8]^ This can be beneficial in enhancing hip joint control^[9]^ and explains the clinical relevance of knee bracing exercises in strengthening core stability and lower extremity stabilizing muscles.

KP and HKP may be considered to be more unstable than SP because they are unnatural positions.^[1]^ In particular, when in SP, the center of pressure (COP) can be shifted forward towards the front of the ankle joint. However, in the KP, it is not possible to shift the COP forward towards the front of the knee joint.^[9]^ In a previous study, the COP of the sagittal plane was analyzed in the SP and KP, and the observed difference in the COP between the 2 conditions was attributed to neural processes and biomechanical factors.^[1]^ In another study, the muscle activities of healthy participants were compared between the KP and SP muscles during trunk flexion exercises. Despite similar trunk muscle activity, it was found that the extensor capability was decreased in the KP compared to that in the SP.^[10]^

Research on the activity of the lower extremity muscles is lacking compared to the ergonomic aspects^[11]^ and trunk muscle activity^[10]^ of KP and HKP. Therefore, the purpose of this study was to investigate the effect of squats in KP and HKP on lower extremity muscle activity to provide essential data for future exercise prescriptions. The hypothesis of this study was that lower extremity muscle activity would vary depending on the squat position.

## 2. Materials and methods

### 2.1. Study design, setting, and participants

This study is a cross-sectional study and selected 30 participants who met the inclusion criteria. In this study, convenience sampling was used to recruit participants. Participants were recruited by posting a notice on the bulletin board of Soonchunhyang University Hospital, Gyeonggi Province, targeting hospital employees. All experiments were conducted in the physical therapy department at Soonchunhyang University Hospital. The recruitment period for participants was 15 days, and the experiment lasted 20 days, totaling 35 days. The average exposure time for participants was 30 minutes.

The inclusion criteria for the participants were healthy adults aged 20 to 60 years, individuals who had not experienced knee pain in the past 6 months, and those without any orthopedic disorders in any part of the lower extremities. The exclusion criteria for participants were individuals with neurological disorders or conditions such as cancer or pregnancy that would hinder their ability to perform the exercises.^[8]^

The sample size of the experimental participants was determined using the G-Power software (ver. 3.1.9.7; Heinrich Heine University, Dusseldorf, Germany), based on data from previous studies. An F test (repeated measures ANOVA) was employed with an effect size of 0.25, statistical power of 0.8, and a significance level (α level) of 0.05. The total sample size was 30 participants.

### 2.2. Study instruments

The study targeted 30 healthy adults, and the participants’ general characteristics, such as sex, age, height, and weight, were recorded before any evaluation. The participants performed squats in 3 different positions as a single group. To eliminate the learning effect, the movements were performed in a random order, and to prevent muscle fatigue between measurements, a 1 minute rest was provided between each movement.

Before the experiment, the maximal voluntary isometric contraction (MVIC) of each muscle was measured. To prevent muscle fatigue, a 5 minute rest was provided and the participants practiced each squat. Subsequently, the participants performed squats in the SP, KP, and HKP. The evaluation was conducted using surface electromyography (EMG) with the EMG electrode placement sites being the rectus femoris (RF), gluteus maximus (GMax), gluteus medius (GMed), and biceps femoris (BF). EMG was performed thrice for each muscle, and the mean value was used as the final result. The experiment was conducted in the physical therapy room at Soonchunhyang University Hospital.

**Figure 1.**
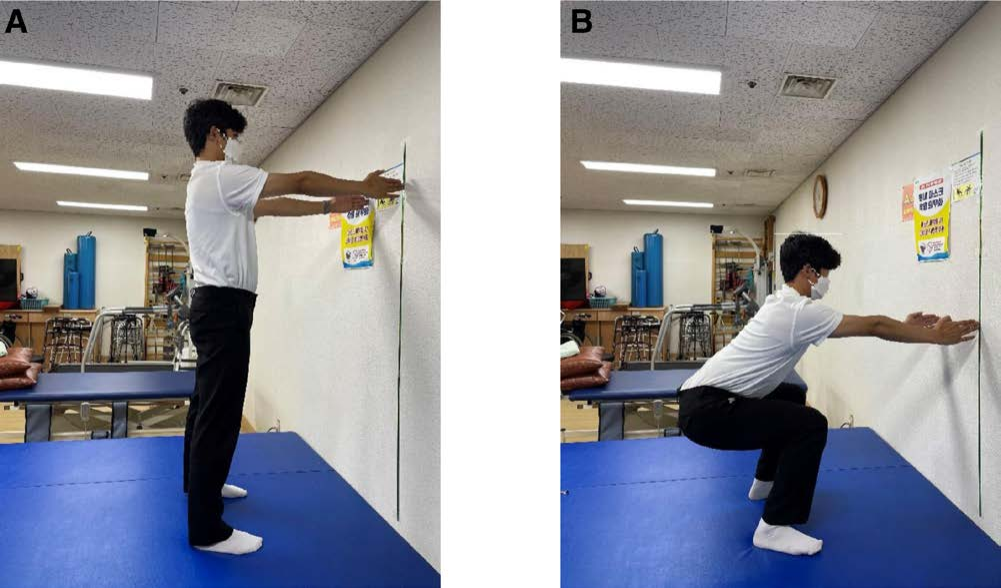
Squat in the standing position. (A) Starting position for the squat in the standing position. (B) Ending position for the squat in the standing position.

**Figure 2.**
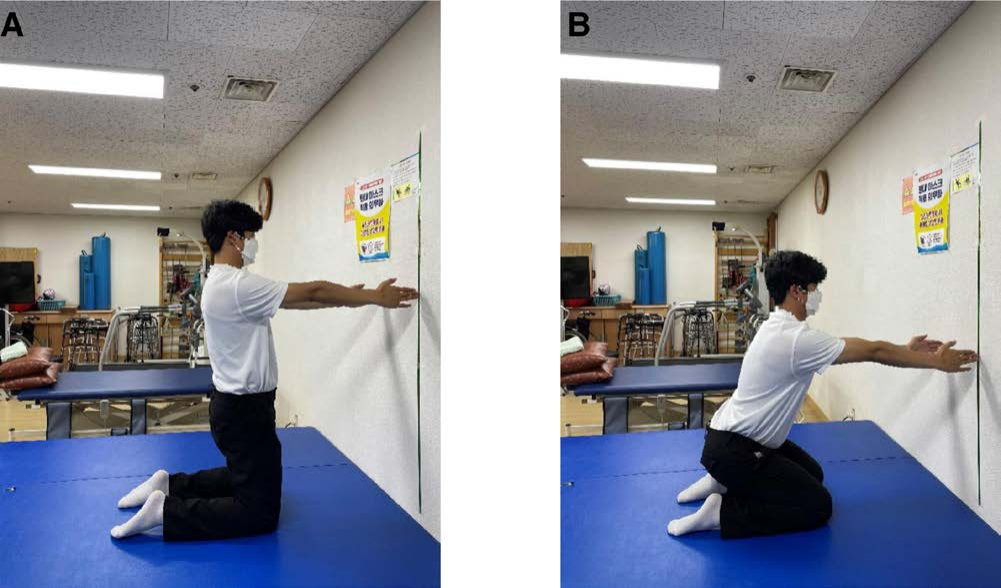
Squat in the kneeling position. (A) Starting position for the squat in the kneeling position. (B) Ending position for the squat in the kneeling position.

**Figure 3.**
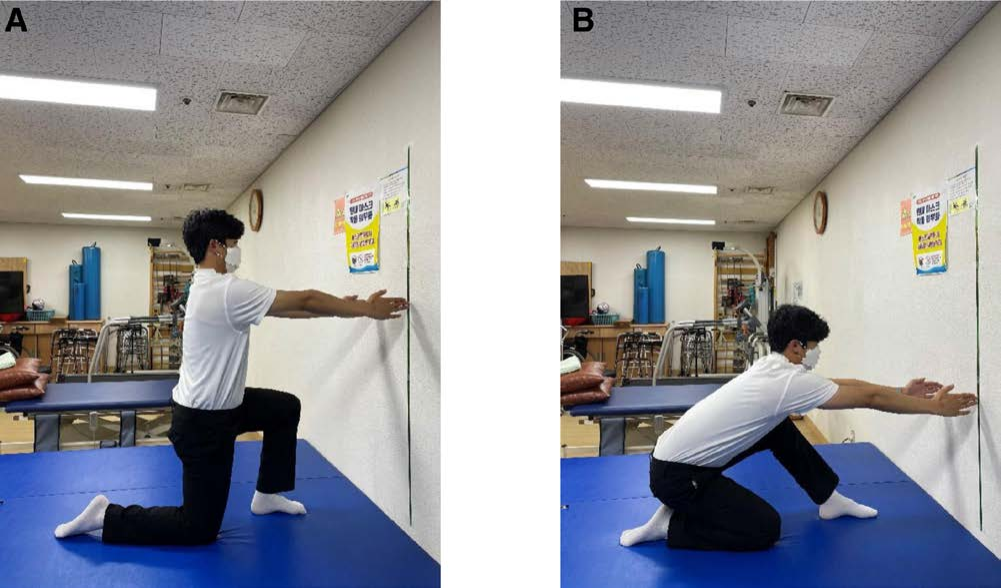
Squat in the half-kneeling position. (A) Starting position for the squat in the half-kneeling position. (B) Ending position for the squat in the half-kneeling position.

Squats from an SP were performed with the feet positioned shoulder-width apart and the acromion process and lateral malleolus aligned vertically (Fig. 1A). For the squats, the knee flexion angle was set at 90° (Fig. 1B). Squats from a KP were performed with the knees positioned hip-width apart, and the acromion process aligned with the femur to create a vertical line (Fig. 2A). The knee and ankle joints were positioned in a straight line so that they were parallel. For squats, a certain degree of knee flexion was set to prevent the buttocks from touching the heels (Fig. 2B). Squats from an HKP were performed by starting in the KP, the opposite leg was extended forward, and the foot was positioned to create a 90° angle between the hip and knee joints (Fig. 3A). For squats, a certain degree of knee flexion was set to prevent the buttocks from touching the heels (Fig. 3B).

All 3 positions maintained with the participant facing forward with the fingertips extended to touch the guideline mounted perpendicular to the wall. Additionally, a 1 cm thick mat was used to prevent knee discomfort during the squats. The participants performed squats for 9 seconds, with 4 seconds in the descent phase, 1 second in the maintenance phase, and 4 seconds in the ascent phase, using a metronome to ensure precise timing. To minimize muscle fatigue, 1 minute rest period was provided between each test. The participants experimented with sufficient preliminary practice to familiarize themselves with the squatting movements before the actual testing.

### 2.3. Outcome measures

Surface EMG (Ultium EMG^®^; Noraxon, Scottsdale) was used to measure muscle activity. To minimize skin resistance before electrode attachment, bodily hair was removed from the site and was cleaned with alcohol swabs. The signal was rectified by obtaining its absolute value. All test data were normalized by dividing each muscle’s value by its corresponding MVIC. Disposable self-adhesive Ag/AgCl dual snap surface electrodes (Noraxon, Scottsdale) with a center-to-center electrode spacing of 2 cm were utilized. The EMG signals were processed as follows: the sampling rate was set to 1500 Hz, and the frequency range was set to 20 to 500 Hz, and root mean square was configured for 300 ms.^[8]^ The analysis was performed using the MR3 software (Noraxon, Scottsdale).

The RF electrode was attached midway between the center of the patella and the anterior superior iliac spine. The GMax electrode was attached obliquely, parallel to the muscle fibers, 3 cm from the greater trochanter to the midpoint of the sacrum, at the height of the greater trochanter. The GMed electrode was attached parallel to the muscle fibers, with a 2 cm distance between them, at 1/3rd of the distance between the iliac crest and the greater trochanter. The BF electrode was attached 2 cm at the midpoint in the posterior aspect of the femur between the gluteal sulcus and patella, parallel to the muscle fibers.^[12]^

The dominant leg of all subjects was measured, and the positioning and angles of each muscle were based on the manual muscle tests. The RF was measured in the supine position, GMed was measured in the side lying position, and GMax and BF were measured in the prone positions.^[13]^ To normalize (%MVIC) the data, we used EMG signals from the 6 second segment encompassing the ascent and descent phases of the 9 second squat, excluding the maintenance phase time. Using these values, we compared the muscle activity levels between the 3 different positions.

The co-contraction ratio (CCR) indicates the ratio of normalized agonistic muscle activity to normalized antagonistic muscle activity.^[14]^ The CCR value is related to smooth movement and coordination. In this study, the criteria for defining agonists and antagonists were based on anatomical movements, rather than the degree of muscle contraction. The RF was designated as an agonist, whereas the Gmax and BF were classified as the antagonists. The CCR was calculated using the following equation:^[15]^


$$\begin{array}{l} CCR=\;\frac{sEMG\left(antagonistic\;\;\;muscle\right)}{sEMG\left(agonistic\;muscle\right)+sEMG\left(antagonistic\;\;\;muscle\right)} \end{array}$$

### 2.4. Descriptive statistics

All statistical analyses and procedures were performed using SPSS Statistics for Windows, version 22.0 (IBM Corp., Armonk). The participants’ general characteristics were described using descriptive statistics. To compare the effects of the 3 squat positions on lower extremity muscle activity, the 1-way repeated measures ANOVA was conducted. If the interaction was significant, a post hoc test using the Least Significant Difference (LSD) method was conducted. Statistical significance was set at *P* <.05.

### 2.5. Ethical consideration

The recruitment was based on voluntary participation. The participants were provided with an explanation of the experimental procedures and conditions and signed an agreement to participate in the study before commencement. This study received approval from the Institutional Review Board (IRB) of Sahmyook University (approval no. 2023-04-010-002). Registered with the Clinical Research Information Service, this study adheres to the World Health Organization International Clinical Trials Registry Platform (WHO-ICTRP) guidelines (registration number: KCT0008905).

## 3. Results

The study included 30 participants (19 males and 11 females), with no dropouts. The mean age was 35.5 years, the mean height was 170.4 cm, and the mean weight was 67.6 kg (Table 1).

**Table 1:** General characteristics of subjects (N = 30).

	Subjects
Gender	
Male	19
Female	11
Age (yr)	35.5 ± 8.2
Height (cm)	170.4 ± 8.4
Weight (kg)	67.6 ± 13.3

Values are presented as number or mean ± standard deviation.

**Figure 4.**
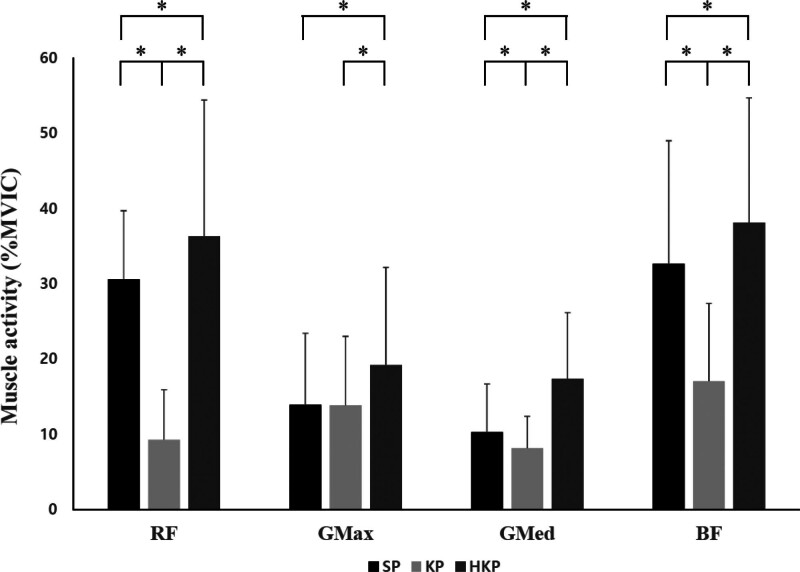
Changes in muscle activity for each position during squats. BF = biceps femoris, GMax = gluteus maximus, GMed = gluteus medius, HKP = half-kneeling position, KP = kneeling position, MVIC = maximal voluntary isometric contraction, RF = rectus femoris, SP = standing position; *P < .05.

**Figure 5.**
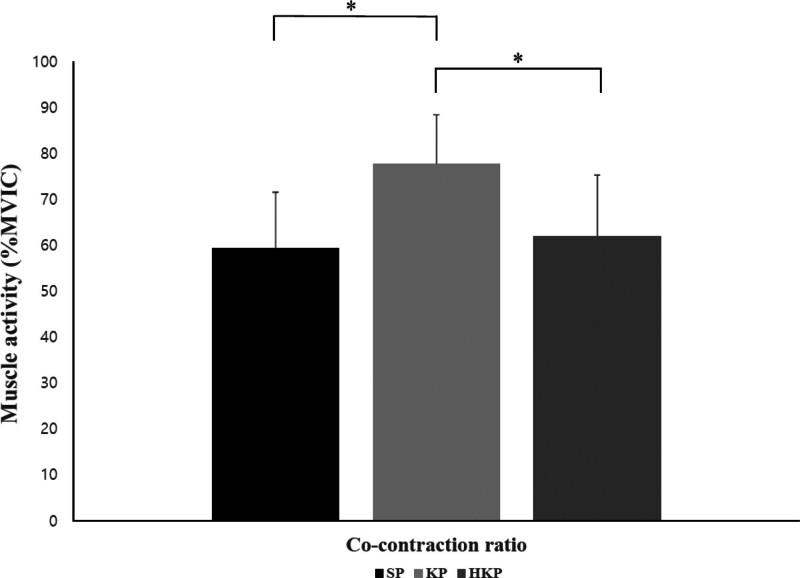
Changes in co-contraction ratio for each position during squats. HKP = half-kneeling position, KP = kneeling position, MVIC = maximal voluntary isometric contraction, SP = standing position; *P < .05.

The muscle activities of the RF, GMed, and BF showed statistically significant differences among the 3 positions, highest in the HKP, followed by the SP, and then the KP (*P* < .05). The muscle activity of the GMax was significantly higher in the HKP than in the SP and KP (*P* < .05; Table 2, Fig. 4). CCR was significantly higher in the KP than in the SP and HKP (*P* < .05), whereas there was no statistically significant difference between the SP and HKP (Fig. 5).

**Table 2:** Muscle activity in each position during squats (N = 30).

	SP (%MVIC)	KP (%MVIC)	HKP (%MVIC)	*F*	*P*	Post-hoc
RF	30.6 ± 9.1	9.26 ± 6.66†	36.34 ± 18.09†,‡	172.194	<.001*	HKP SP KP
GMax	13.92 ± 9.5	13.86 ± 9.15	19.22 ± 12.95†,‡	4.432	.021*	HKP SP, KP
GMed	10.31 ± 6.37	8.16 ± 4.22†	17.37 ± 8.8†,‡	20.215	<.001*	HKP SP KP
BF	32.69 ± 16.34	17.07 ± 10.32†	38.13 ± 16.58†,‡	48.34	<.001*	HKP SP KP
CCR	59.36 ± 12.24	77.76 ± 10.7†	62.05 ± 13.28‡	51.265	<.001*	KP SP, HKP

Values are presented as mean ± standard deviation.

BF = biceps femoris, CCR = co-contraction ratio, GMax = gluteus maximus, GMed = gluteus medius, HKP = half-kneeling position, KP = kneeling position, MVIC = maximal voluntary isometric contraction, RF = rectus femoris, SP = standing position.

* *P* < .05.

† Conditions that showed a significant difference from SP.

‡ Conditions that showed a significant difference from KP.

**Figure 6.**
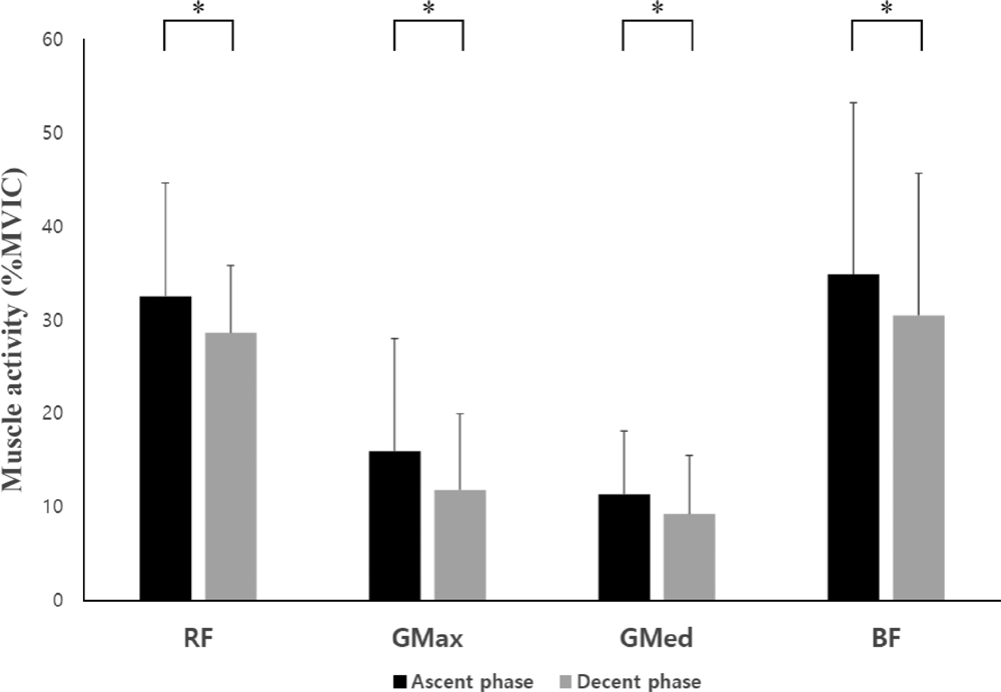
Changes in muscle activity for each phase during squats in the supine position. BF = biceps femoris, GMax = gluteus maximus, GMed = gluteus medius, MVIC = maximal voluntary isometric contraction, RF = rectus femoris; ^*^P < .05.

**Figure 7.**
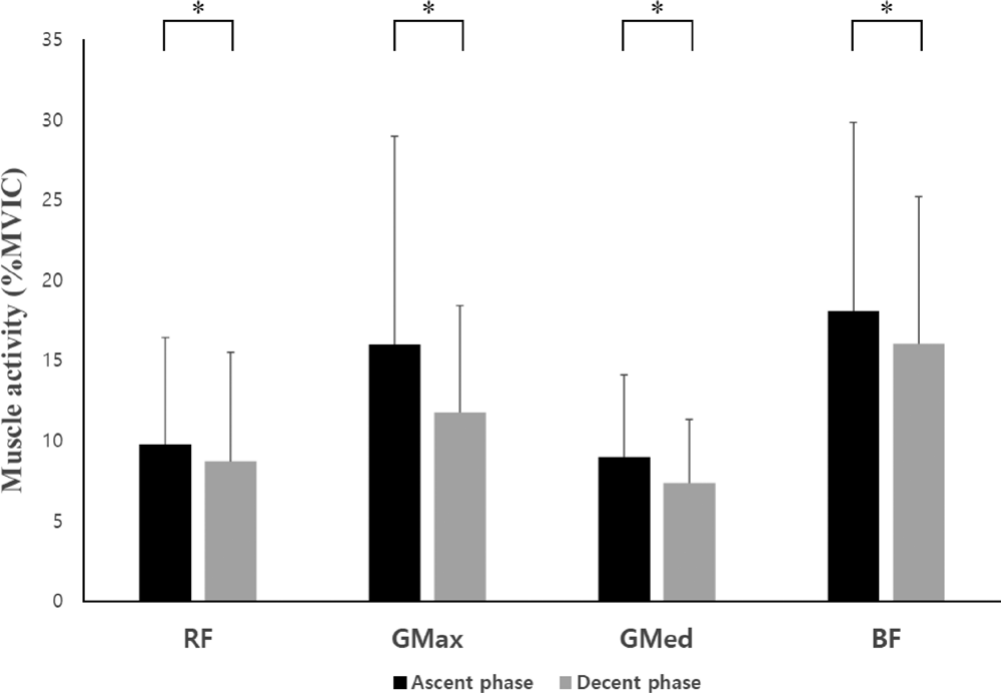
Changes in muscle activity for each phase during squats in the kneeling position. BF = biceps femoris, GMax = gluteus maximus, GMed = gluteus medius, MVIC = maximal voluntary isometric contraction, RF = rectus femoris; ^*^P < .05.

**Figure 8.**
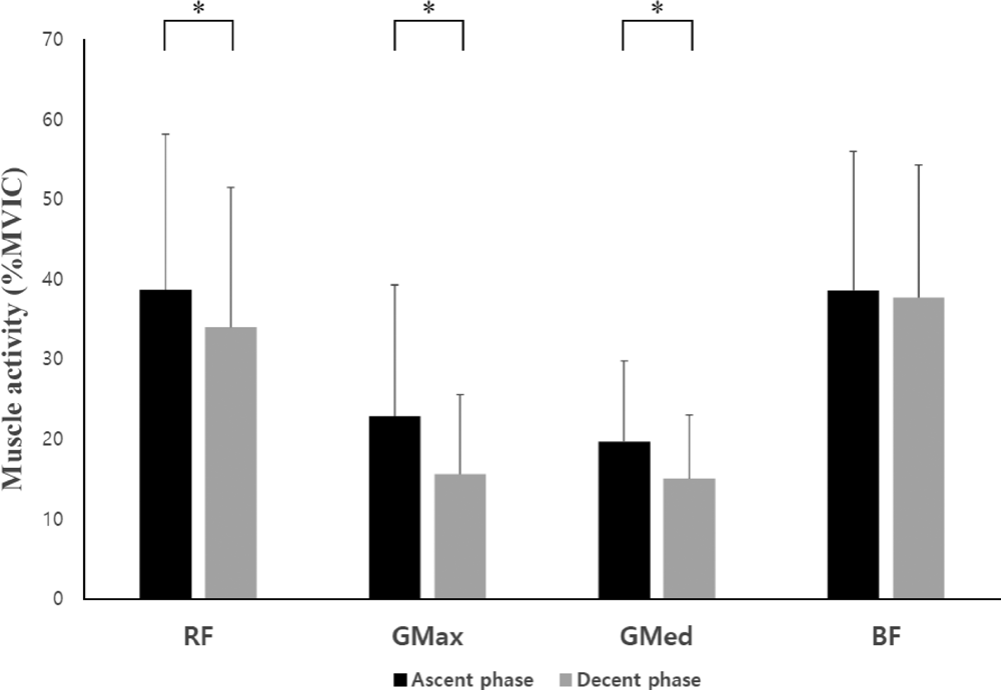
Changes in muscle activity for each phase during squats in the half-kneeling position. BF = biceps femoris, GMax = gluteus maximus, GMed = gluteus medius, MVIC = maximal voluntary isometric contraction, RF = rectus femoris, ^*^P < .05.

In the SP and KP, there were statistically significant differences between the ascent and descent phases of the RF, GMax, GMed, and BF (*P* < .05; Table 3, Figs. 6 and 7). In the HKP, there were statistically significant differences between the ascent and descent phases of the RF, GMax, and GMed (*P* < .05; Fig. 8).

**Table 3:** Muscle activation according to each phase during squats (N = 30).

	SP (%MVIC)		KP (%MVIC)		HKP (%MVIC)	
	Ascent phase	Decent phase	Ascent phase	Decent phase	Ascent phase	Decent phase
RF	32.54 ± 12.11	28.65 ± 7.18*	9.79 ± 6.65	8.72 ± 6.8*	38.68 ± 19.46	34 ± 17.5*
GMax	15.99 ± 12.01	11.86 ± 8.1*	16.01 ± 12.96	11.76 ± 6.66*	22.86 ± 16.41	15.58 ± 9.97*
GMed	11.36 ± 6.79	9.26 ± 6.26*	8.98 ± 5.12	7.35 ± 3.99*	19.67 ± 10.08	15.06 ± 7.95*
BF	34.88 ± 18.37	30.5 ± 15.18*	18.09 ± 11.75	16.05 ± 9.17*	38.57 ± 17.4	37.7 ± 16.6

Values are presented as mean ± standard deviation.

BF = biceps femoris, CCR = co-contraction ratio, GMax = gluteus maximus, GMed = gluteus medius, HKP = half-kneeling position, KP = kneeling position, MVIC = maximal voluntary isometric contraction, RF = rectus femoris, SP = standing position.

* *P* < .05.

## 4. Discussion

Muscle activity of the RF, GMed, and BF significantly increased in the order of HKP, SP, and KP (*P* < .05). A previous study reported that measures of isometric and isokinetic contractions, such as stability and strength, were significantly decreased in the KP compared to the SP.^[10]^ This can be attributed to the increased stability resulting from decreased postural instability owing to changes in the biomechanical system when the COP is closer to the ground.^[1]^ In particular, compared to the KP, the HKP has a smaller base of support (BOS), and the SP has an even smaller BOS, with a higher COP. This results in reduced postural stability, necessitating an increased demand for force to control body posture. As the COP approaches the ground, changes in the biomechanical system result in postural instability.^[1]^ Nevertheless, in a previous study, trunk muscle activity was similarly measured.^[10]^ This result suggests that to achieve forces equivalent to the SP, the trunk muscles in the KP and HKP need to generate stronger activation.

The muscle activity of the GMax was significantly increased in the HKP compared to that in the SP and KP (*P* < .05), but there was no statistically significant difference between the SP and KP. According to a previous study, the muscle activity of the GMax during kneeling gait was significantly higher than in normal walking.^[2]^ These findings demonstrate that despite increased stability from the KP, there is an increase in proximal muscle activity. In the case of the proximal region with many single joint muscles, compared to the distal region with many multi joint muscles, this is hypothesized to be an adaptation to assist in stabilizing the trunk muscles to achieve the equivalent strength of an upright posture, leading to a reduction in the number of muscles aiding the movement.^[16]^ In other words, it can be observed that the muscle activity of GMax increased in the KP to prevent trunk sway and enhance stability.

Additionally, in both SP and KP, the muscle activity of the RF, GMax, GMed, and BF was higher during the ascent phase than during the descent phase (*P* < .05). Similarly, the HKP, RF, GMax, and GMed also exhibited higher muscle activity during the ascent phase than during the descent phase (*P* < .05). In contrast, in the HKP, the BF was not significantly different between the ascent and descent phases (*P* > .05). This suggests that in future research, it may be necessary to examine the peak values of muscle activity rather than the mean values.

Compared to isometric and concentric contractions, eccentric contractions can generate a greater force.^[17]^ During eccentric contractions, the sarcomere length increases, including the active stretch of the spring (structures such as the titin myofilament), increasing its stiffness.^[18]^ The magnitude of the eccentric contraction force depends on the measurement conditions, with a potential increase in the force/moment of up to 30% in single joint movements^[19]^ and up to 10% in multi joint movements.^[20]^ These differences are likely attributable to specific neural activation strategies during eccentric contractions and variations in neural activation during multi joint movements.^[21]^ In traditional squats, the ground reaction force is reportedly greater during the concentric phase than the eccentric phase.^[22]^ It has been reported that joint moments during the concentric phase are 11% to 20% greater than those during the eccentric phase.^[23]^ These findings are consistent with the electromyography results of our study. However, unlike previous studies, in our study, it appears that as the trunk leaned forward, the moment arm increased, leading to higher muscle activity in the hip joint.

CCR is a functional parameter that signifies muscular balance and coordination and involves the simultaneous activation of agonist and antagonist muscles in static or dynamic states.^[24]^ A high CCR indicates greater activity of the GMax and BF muscles compared to the RF, whereas a low CCR signifies lower activity of the GMax and BF muscles compared to the RF. In 1 study, the CCR was highly correlated with trunk moment.^[25]^ These findings suggest a strong association between trunk stiffness and stability. However, another study demonstrated that despite a consistent trunk moment, an increasing need for biomechanical stability leads to a higher CCR.^[26]^ In other words, the CCR indicates an increase in the potential energy of the biomechanical system to maintain stability, regardless of the trunk moment. In this study, the CCR was the highest in the KP, followed by the HKP and SP. These results suggest that an increase in CCR is likely to enhance body stability. A higher CCR can be attributed to the elevated activation of antagonistic muscles, which, in turn, contributes to greater body stability. Specifically, the ratio of BF to RF muscle activity was higher in the KP than in the other positions. This increase in muscle activity can be seen as a compensatory strategy through concentric contraction of the BF to reduce patellofemoral pressure^[27]^ or as an attempt to enhance knee joint stiffness.^[28]^

The results of this study suggest that varying the BOS and COP heights provided by different positions can influence lower extremity muscle activity. The base of the SP is composed of only the soles of the feet and is significantly smaller than that of the KP.^[8]^ The increased stability during KP, when compared to the SP, appears to have reduced the demand for muscle strength in the thigh muscles, including those stabilizing the hip and knee joints. On the other hand, in the HKP, the BOS is wider than that in the SP; however, complete weight bearing on 1 leg appears to increase the demand for strength in the leg muscles.

The HKP possesses a BOS that is nearly half that of the KP, and it has been reported that muscle activity increases compared with the position where both knees are on the ground.^[8]^ It should also be noted that the raised knee forms a barrier that causes a shift in the load.^[29]^ As a result, an increased load moment may have contributed to the higher thigh muscle activity. A KP can be considered an unnatural position and may be perceived as less stable than an SP.^[1]^ However, the BOS in the SP was composed only of the soles of the feet and was significantly smaller than those in the other studied postures. It could be considered the most unstable posture among the studied positions. The knees and pelvis are positioned in unsupported locations, increasing the need to activate the lower extremity muscles to maintain postural stability.

In the KP, ankle movement is restricted, whereas in the HKP, ankle mobility is limited because of minimal weight bearing. In other words, in the KP and HKP, it becomes challenging to utilize the inherent proprioceptive input from the soles of the feet and ankles for balance control. In conclusion, to maintain balance, dependence is placed on somatosensory inputs originating from structures associated with the visual, vestibular, and knee joint systems.^[1]^ These findings suggest that, when applied to neurological patients, the KP and HKP may enhance somatosensory perception related to the knee joints compared to the SP. In the KP and HKP, the lower COP and wider BOS compared to standing resulted in a decreased risk of falling. Additionally, there is an advantage that can eliminate the impact of ankle strategies, which inevitably affect other joints. Patients with stroke often rely on hip strategies for postural control because of ankle function deficiencies. Therefore, positions such as the KP and HKP are highly beneficial.^[30]^

This study confirmed that squats performed in the HKP are advantageous for increasing the overall muscle activity in the lower extremities. Furthermore, the order of effectiveness in facilitating overall muscle activity was the HKP, SP, and KP. In addition, the KP is applicable when lower intensity squats are required. However, prolonged maintenance of the KP for more than 15 minutes can lead to low back pain,^[16]^ and substantial external forces are exerted on the knees during activities performed in this position.^[31]^ Previous studies have reported that the KP demands a greater knee flexion angle than the SP.^[32]^ A greater knee flexion angle should be approached with caution because it can induce greater moments at the knee joint.^[33]^

This study had several strengths and limitations. Strengths include the use of electromyography, a widely validated measure, and the analysis using robust statistical methods, ensuring the accuracy and validity of the results. Additionally, the findings were easily interpretable, providing clear insights into the research subject. However, the relatively small sample size may limit the generalizability of the findings, and despite controlling for multiple confounding variables, there may still be unmeasured or overlooked confounders that could affect the results.

## 5. Conclusions

The results of this study indicate that squats in the HKP (especially during the ascent phase) require the highest muscle activity, whereas squats in the KP (especially during the descent phase) can be performed with the lowest muscle activity. Despite the lower muscle activity in the KP, the CCR was higher, which suggests relatively greater activation of the antagonist muscles compared to the agonist muscles. These findings are considered to be potentially useful indicators for the selection of squat positions and for the enhancement of postural control, muscle strength, and lower extremity stabilization.

## Acknowledgments

This research received no specific grant from any funding agency in the public, commercial, or not-for-profit sectors.

## Author contributions

**Conceptualization:** Taewoong Jeong, Yijung Chung.

**Data curation:** Taewoong Jeong, Yijung Chung.

**Formal analysis:** Taewoong Jeong, Yijung Chung.

**Investigation:** Taewoong Jeong.

**Methodology:** Taewoong Jeong, Yijung Chung.

**Project administration:** Taewoong Jeong.

**Resources:** Taewoong Jeong.

**Supervision:** Taewoong Jeong, Yijung Chung.

**Validation:** Taewoong Jeong, Yijung Chung.

**Visualization:** Yijung Chung.

**Writing – original draft:** Taewoong Jeong.

**Writing – review & editing:** Taewoong Jeong, Yijung Chung.
